# Dengue, chikungunya, and scrub typhus are important etiologies of non-malarial febrile illness in Rourkela, Odisha, India

**DOI:** 10.1186/s12879-019-4161-6

**Published:** 2019-07-03

**Authors:** Pavitra N. Rao, Anna Maria van Eijk, Sandhya Choubey, Syed Zeeshan Ali, Aditee Dash, Punam Barla, Rajshri Rani Oraon, Gautam Patel, P. Nandini, Subrata Acharya, Sanjib Mohanty, Jane M. Carlton, Sanghamitra Satpathi

**Affiliations:** 1 Center for Genomics and Systems Biology, Department of Biology, New York University, New York, NY USA; 2 Center for the Study of Complex Malaria in India, Ispat General Hospital, Rourkela, Odisha India; 3 Department of Pathology, Ispat General Hospital Rourkela, Rourkela, India; 4 Present Address: Memorial Sloan Kettering Cancer Center, 1275 York Avenue, New York, NY USA

**Keywords:** Febrile illness, Malaria, Dengue, Chikungunya, Scrub typhus, India, Surveillance

## Abstract

**Background:**

We conducted a diagnostic surveillance study to identify *Plasmodium*, dengue virus, chikungunya virus, and *Orientia tsutsugamushi* infections among febrile patients who underwent triage for malaria in the outpatient department at Ispat General Hospital, Rourkela, Odisha, India.

**Methods:**

Febrile patients were enrolled from January 2016–January 2017. Blood smears and small volumes or vacutainers of blood were collected from study participants to carry out diagnostic assays. Malaria was diagnosed using rapid diagnostic tests (RDT), microscopy, and PCR. Dengue, chikungunya, and scrub typhus infections were identified using rapid diagnostic test kits and ELISA.

**Results:**

Nine hundred and fifty-four patients were prospectively enrolled in our study. The majority of patients were male (58.4%) and more than 15 years of age (66.4%). All 954 enrollees underwent additional testing for malaria; a subset of enrollees (293/954) that had larger volumes of plasma available was also tested for dengue, chikungunya and scrub typhus by either RDT or ELISA or both tests. Fifty-four of 954 patients (5.7%) were positive for malaria by RDT, or microscopy, or PCR. Seventy-four of 293 patients (25.3%) tested positive for dengue by either RDT or ELISA, and 17 of 293 patients (5.8%) tested positive for chikungunya-specific IgM by either ELISA or RDT. Ten of 287 patients tested (3.5%) were positive for scrub typhus by ELISA specific for scrub typhus IgM. Seventeen patients among 290 (5.9%) with results for ≥3 infections tested positive for more than one infection. Patients with scrub typhus and chikungunya had high rates of co-infection: of the 10 patients positive for scrub typhus, six were positive for dengue (*p* = 0.009), and five of 17 patients positive for chikungunya (by RDT or ELISA) were also diagnosed with malaria (*p* < 0.001).

**Conclusions:**

Dengue, chikungunya and scrub typhus are important etiologies of non-malarial febrile illness in Rourkela, Odisha, and comorbidity should be considered. Routine febrile illness surveillance is required to accurately establish the prevalence of these infections in this region, to offer timely treatment, and to implement appropriate methods of control.

**Electronic supplementary material:**

The online version of this article (10.1186/s12879-019-4161-6) contains supplementary material, which is available to authorized users.

## Background

Fever is a condition frequently reported by patients using general medicine outpatient departments in Indian hospitals [1, 2]. When a patient complains of fever, additional clinical symptoms are correlated for diagnosis and treatment. Symptoms are often nonspecific, and delays or inaccuracies in arriving at a clinical diagnosis can lead to inappropriate treatment that may result in fatalities. To establish a specific diagnosis and provide the appropriate treatment regimen, diagnoses of febrile illnesses should be confirmed by laboratory tests [3].

Arthropod-borne infectious diseases such as malaria, dengue, chikungunya, and scrub typhus are significant etiologies of acute febrile illness in India [4, 5]. Malaria is caused by mosquito-borne *Plasmodium* parasites, with two species, *P. vivax* and *P. falciparum,* being responsible for the majority of morbidity and mortality. *Plasmodium* parasites alternate between human and *Anopheles* mosquito hosts. Human infection with *Plasmodium* parasites can cause mild to severe illness, encompassing a wide variety of symptoms including fever, headache, malaise, anemia, sweats, and chills that first appear 7–10 days after a bite from an infected mosquito. Left untreated, symptoms can lead to multi-organ failure, severe anemia, cerebral malaria, coma, and death.

Dengue and chikungunya are viral infections transmitted by the bite of infected *Aedes* mosquitoes. The dengue virus (DENV) has an incubation of 4–10 days and can cause disease presentation ranging from asymptomatic to severe. Mild ‘dengue fever’ infections are characterized by fever with or without rash, while more severe symptoms include high fever, severe headache with eye pain, myalgia, arthralgia, and rash. Some patients progress to Dengue Hemorrhagic Fever/Dengue Shock Syndrome (DHF/ DSS), which can be fatal. Four distinct serotypes of DENV can cause the disease, and while infection by any one serotype can lead to lifelong immunity against that serotype, it does not lead to long-term protection against the other serotypes; instead, a subsequent infection by a different serotype has a higher risk of developing into severe disease [6, 7].

Chikungunya is caused by a virus (CHIKV) that has an incubation period ranging from 1 to 12 days following the bite of an infected *Aedes* mosquito. The onset of the disease is characterized by an acute febrile illness, accompanied by rash and severe joint pain that can persist for months [8]. Other symptoms include muscle pain, headache, nausea, and fatigue. Symptoms are often mild in infected individuals and thus the infection may go unrecognized or be misdiagnosed.

Scrub typhus is an acute febrile illness caused by the obligate intracellular bacterium *Orientia tsutsugamushi*. It is transmitted to humans through the bite of the trombiculid mite, which is both the vector and reservoir for the bacterium. Humans are incidental hosts [9]. Scrub typhus is distinguished by the formation in a subset of patients of a scab-like lesion called an ‘eschar’ at the site of mite feeding. Scrub typhus is often under-diagnosed as its nonspecific clinical features, including high fever, lymphadenopathy, rash, myalgia, and headaches, make it difficult to differentiate from other febrile illnesses.

While there is an existing integrated disease surveillance program in India [10], the true burden of these diseases is likely to be higher than reported [11–13], and may vary by region. In addition, delayed or inaccurate diagnoses are more likely in populations living in inaccessible (e.g., rural or remote) areas of India, where cost and a lack of a well-developed health-care infrastructure are obstacles to the use of sensitive molecular diagnostic tests. Routine surveillance is critical for accurate estimates of disease burden, which in turn drives the implementation of public health policies for prevention, diagnosis, and treatment of common etiologies of infectious disease.

This manuscript describes a pilot project to determine whether chikungunya, dengue, and scrub typhus were common among patients presenting with acute febrile illness who had been triaged for malaria in an outpatient department (OPD) at Ispat General Hospital, Rourkela, in the state of Odisha, India. A second aim of the study was to undertake a comparative assessment of the performance of several standard, commercially available diagnostic tests for these three febrile illnesses. A third aim was to determine if malaria cases were missed during the initial malaria triage by the OPD.

## Methods

### Study site

Rourkela is located in Sundargarh district close to the northern border of the state of Odisha, India, and has a population of over 0.5 million. It has a tropical climate, with high temperatures and heavy rainfall between June–October and December–January. Subjects were enrolled at the outpatient department (OPD) of Ispat General Hospital (IGH), which is a 685-bed hospital for employees of Rourkela Steel Plant, Steel Authority of India, Inc., and for people living in and around Rourkela. A total of 90,867 patients attended the OPD at IGH Rourkela in 2016, of which 13,534 patients (~ 15%) reported fever.

### Study design and inclusion criteria

Between January 2016–January 2017, 954 OPD patients aged 12 months - 70 years (with children categorized as ≤15 years and adults as ≥16 years) who reported having fever up to 48 h prior to enrollment or who were febrile on day of enrollment (body temperature ≥ 37.5 °C) were enrolled in our study. These patients had already been through triage for malaria in the OPD, where probable malaria cases were identified based on presence of high fever, chills, and rigor. Such cases, along with individuals with symptoms of influenza and pregnant women (due to the possible risks associated with enrolling these subjects), were not enrolled in our study. Written consent or assent was obtained from each enrolled patient prior to a physical examination and administration of a detailed clinical questionnaire, and each patient provided a blood sample.

### Sample collection and processing

All 954 enrollees were tested for malaria by at least one diagnostic test; a subset of enrollees (293/954) from whom a larger volume of blood was collected was also tested for dengue, chikungunya, and scrub typhus by either RDT, ELISA, or both tests. Patient blood samples were collected either in a microvette (small volume ~ 100 ul) and/or a vacutainer (large volume ~ 3-5 ml) at the time of enrollment and tested by Hemocue to assess haemoglobin levels. Whole blood was used for thin and thick blood smear preparations, as well as malaria RDTs. Microvette and vacutainer samples were centrifuged to separate plasma and red blood cells, and these fractions were stored at − 80 °C before use. DNA was extracted from the red blood cells using QIAamp DNA blood Mini Kits (Qiagen Inc., Valencia, CA).

### Diagnostic assays

Malaria: Three tests -- rapid diagnostic test (RDT), blood smear microscopy, and polymerase chain reaction (PCR) -- were used for malaria diagnosis. All 954 participants were tested *P. falciparum* and *P. vivax* by RDT (FalciVax, Zephyr Biomedicals, India, a commercially available lateral flow kit that detects *P. falciparum* histidine-rich protein 2 and *P. vivax* lactate dehydrogenase) at time of enrollment. All participants also had a thick and thin blood smear made with whole blood from a finger prick, stained with Giemsa and examined by microscopy using a 100x oil immersion objective. Thick smear parasitemia was calculated against 200–500 leukocytes and expressed as parasites per microliter of blood, using the white blood cell (WBC) count if determined, or assuming 8000 WBCs per microliter blood. A semi-nested PCR assay (modified from [14]) targeting the 18S small subunit ribosomal protein gene (SSU rDNA) was used for species-specific molecular detection of four *Plasmodium* species (*P. falciparum, P. vivax, P. malariae,* and *P. ovale*) using DNA extracted from a subset of patients (852/954, 89.3%) from whom a microvette (~100ul) or vacutainer (~ 3-5 ml) of blood had been collected.

Dengue, chikungunya, and scrub typhus: Testing for dengue, chikungunya, and scrub typhus was done on a subset of patients (293/954, 30.7%) enrolled from July 2016 to January 2017 from whom larger volumes of blood were available (since larger volumes of plasma are required for dengue and chikungunya RDTs). A pilot study was first conducted with blood samples from approximately 100 of these 293 patients, to test two commonly used dengue RDTs (Dengue Day 1 test from J. Mitra and Co., India, and SD Bioline Dengue Duo test from Alere Medical, India) and two chikungunya RDTs (Advantage Chikungunya IgM card RDT from J. Mitra and Co., New Delhi, India; and the SD Bioline Chikungunya IgM RDT from Alere Medical, India) [15–17]. The Dengue Day 1 test and the SD Bioline Dengue Duo test recognize the dengue non-structural protein 1 antigen (NS1) and DENV-specific IgM and IgG antibodies in patient sera. The Advantage Chikungunya IgM card RDT and the SD Bioline Chikungunya IgM RDT detect chikungunya-specific IgM in patient sera. The Dengue Day 1 kit and the Advantage Chikungunya IgM card RDTs, both from J. Mitra and Co., worked more efficiently in our hands and were subsequently chosen for all downstream diagnoses.

Since the sensitivity and specificity of ELISA-based diagnostic kits are better than RDTs, we also used ELISA kits to test extracted plasma samples for dengue and chikungunya. The Panbio Dengue Early ELISA kit for detection of NS1 antigen (Alere Medical, India), and the Panbio Dengue IgM Capture ELISA (Alere Medical, India) were used for diagnosis of dengue, and the Chikungunya IgM ELISA kit (SD Bioline) was used for diagnosis of chikungunya.

The Scrub Typhus Detect IgM ELISA kit (InBios International, Inc., Seattle, WA, USA) was used to detect *Orientia tsutsugamushi*. Optical density values > 0.5 were considered as a positive test result [18, 19].

**Fig. 1:**
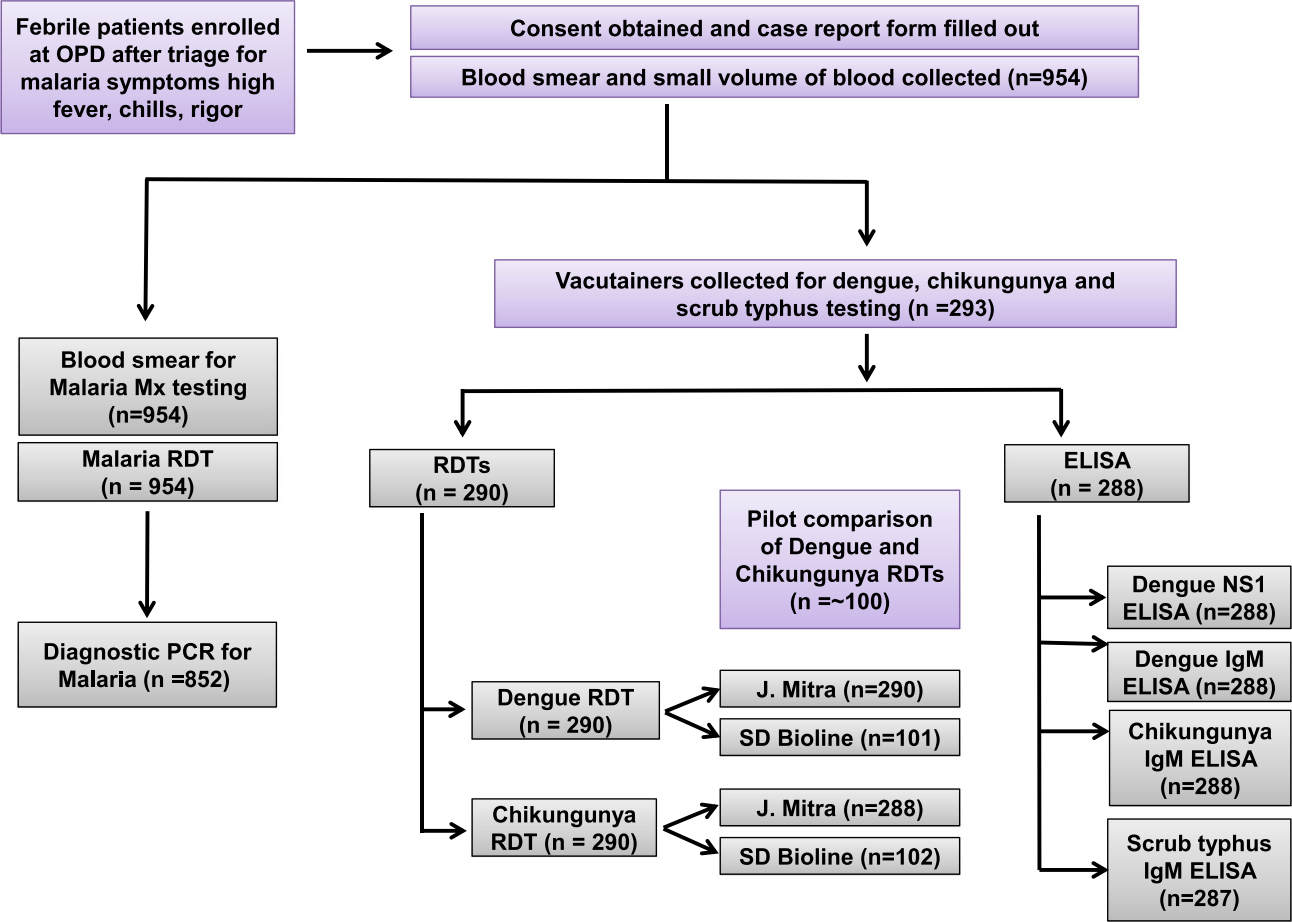
Flowchart of patient testing for febrile illness. A total of 954 patients in the outpatient department at IGH, Rourkela, with reported fever up to 48 h prior to enrollment or on day of enrollment (body temperature ≥ 37.5 °C), were enrolled. All study participants were tested for *Plasmodium* by RDT and microscopy, and a subset of patients was tested by PCR. Patients from whom a larger volume of blood was available were tested for dengue, chikungunya and scrub typhus by RDT and ELISA

A flowchart describing samples tested for malaria, dengue, chikungunya, and scrub typhus using the above diagnostic tests is shown in Fig. 1.

### Data management, definitions, and analysis

Patient clinical and demographic questionnaire data were collected on paper case report forms (CRFs) that were entered and uploaded into the CSCMi’s REDCap database [20]. Results for all assays carried out on plasma, blood, and DNA samples were also uploaded into CSCMi REDCap. Data sets were exported into Stata (version 14.2). A patient was considered to have malaria if any malaria test (RDT, microscopy, or PCR) was positive. Based on national guidelines [21] our case definitions for dengue diagnosis were as follows: 1) patients with NS1 or IgG/IgM positive RDT results were considered probable dengue fever cases, and 2) patients with NS1 or IgM positive ELISA results were considered current or past primary or secondary early dengue infection cases. Patients with IgM positive RDT or ELISA results were considered probable chikungunya cases [22]. Patients with febrile illness, with or without eschar, who had plasma positive for IgM (O.D. > 0.5) by ELISA were considered probable scrub typhus cases [18]. The period from June–October was considered the rainy season; age was categorized into ≤15 (child) and ≥ 16 years (adult). Symptoms evaluated for their association with laboratory results included documented fever (body temperature ≥ 37.5 °C at the time of enrollment), headache, aches, chills, vomiting, diarrhea, cough, dizziness, sweating, abdominal pain, chest pain, eye or ear pain, or rash. The association between laboratory test results and symptoms, age, gender, season, and anemia (for the definition see Additional file 1: Table S1) were examined using the chi-square test in univariate and generalized linear regression models for multivariate models with a log link and binomial distribution [23]. A *p*-value < 0.05 was considered significant. The concordance between diagnostic test results was examined using the McNemar test and visualized using proportional Venn diagrams in Stata (version 14.2).

## Results

### Patient demographics and clinical presentation

**Fig. 2:**
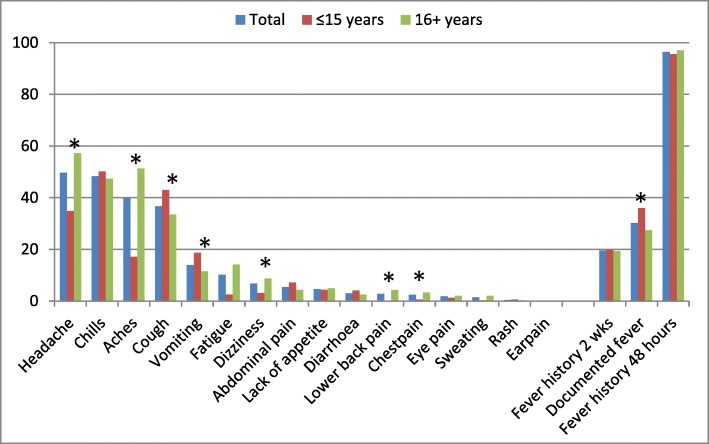
Clinical features of study participants. A graphical distribution of clinical features of study participants in the outpatient department at IGH, Rourkela is presented with features on the x-axis and proportion of patients on y-axis. The patients were categorized as children (15 years and below) and adults (16 years and above), with an asterisk to denote statistically significant differences between children and adults (*p = < 0.05 comparing children vs. adults)

We enrolled 954 participants from patients attending the outpatient clinic at Ispat General Hospital, in Rourkela, Odisha, India from January 2016 to January 2017. A total of 58.4% patients were male and 66.4% > 15 years of age, with a median age of 25 (interquartile range 12–44). Most patients reported having fever in the last 48 h (96.5%) and 276 patients (30.2%) were febrile at the time of enrollment; the use of fever suppressant medication was common (76.6%). The most common clinical symptoms presented by patients included headache (49.7%), chills (48.3%), and aches or pains (39.8%); symptoms differed significantly by age group for eight symptoms, with documented fever, vomiting, and cough more common in children (≤15 years), and chest pain, lower back pain, dizziness, aches, and headache more common among persons aged 16 years and above (Fig. 2). There were no differences in symptoms by gender; documented fever was more common in the rainy season (33.9% vs. 24.9% in dry season, *p* = 0.004) and chills were more common in the dry season (43.6% in rainy vs. 55.5% in dry, *p* < 0.001). Sixty-four patients (6.7%) reported being diagnosed with malaria at least once in the past year, and 58 of them (90.6%) reported receiving antimalarial therapy. Anaemia was detected among 38.8% of the study population.

### Malaria

**Fig. 3:**
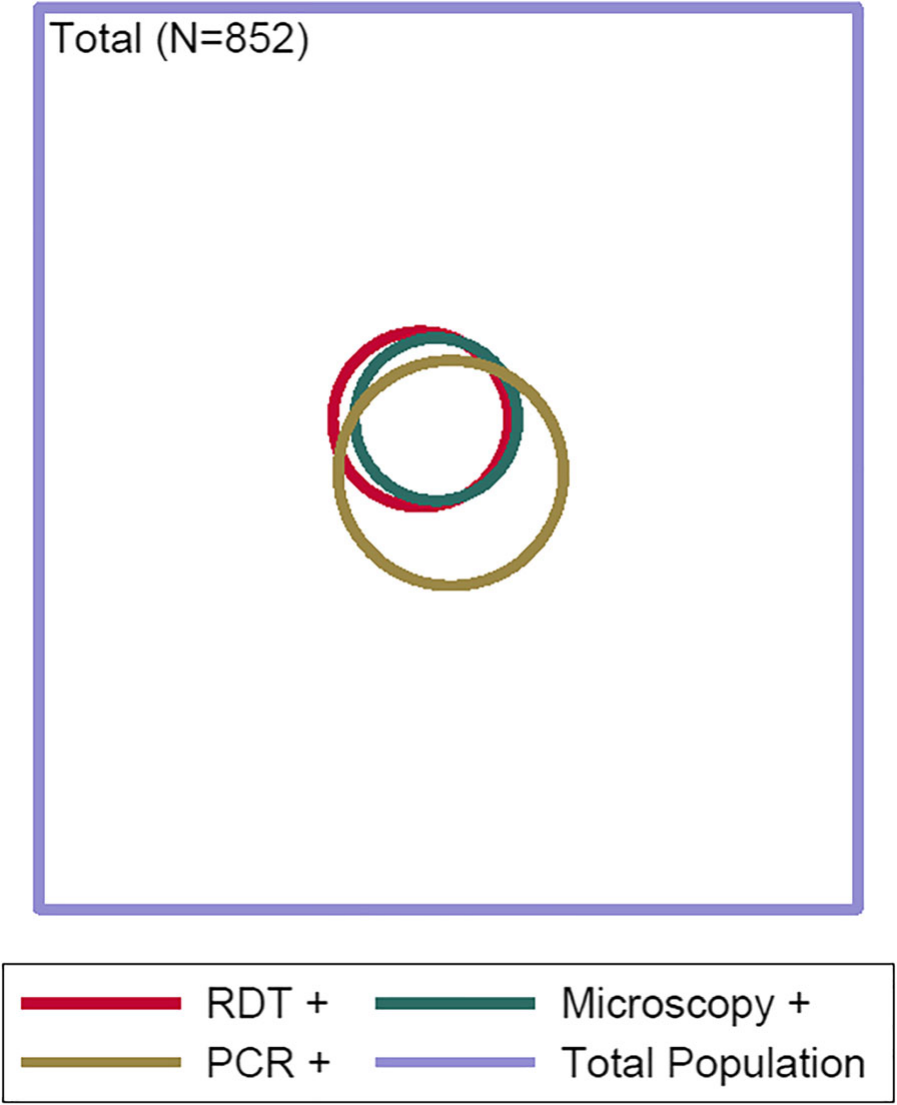
Venn diagram of malaria test results among OPD patients, Rourkela, 2016–2017. A proportional Venn diagram representing *Plasmodium* positivity among 852 study participants each tested by three different assays: RDT, microscopy and PCR. Each circle represents positives by the different assays, with the numbers also shown in Table 2

Fifty-four out of 954 patients (5.7%) were positive for *P. falciparum*, *P. vivax,* or *P. malariae* by at least one diagnostic method (RDT, microscopy, or PCR): 46 by PCR (46/852, 5.4%), 30 by RDT (30/954, 3.1%) and 25 by microscopy (25/954, 2.6%; Table 1). Among the 852 patients tested by all three tests, three patients were positive by RDT but not by another malaria test: two of these (both with *P. falciparum*) had a history of antimalarial treatment within the last month, but this was absent in the third patient who had both *P. falciparum* and *P. vivax* (Table 2). No patient was positive by microscopy and negative by both other tests (Table 2). Three patients were positive by RDT and microscopy but not by PCR, with species matching (2 *P. vivax* and 1 *P. falciparum*). Twenty-three patients were positive by PCR but not by RDT or microscopy (sub-patent infections, all *P. falciparum*); when only considering microscopy, 25 patients had submicroscopic infections (positive by PCR, but negative by microscopy, 1 *P. vivax*, and 24 *P. falciparum,* one of each was also detected by RDT). A proportional Venn diagram (Fig. 3) shows the concordance between the malaria diagnostic test results.

**Table 1:** *Plasmodium* infections among OPD patients as determined by RDT, microscopy and PCR, Rourkela 2016–2017

Test	Total tested	*P. falciparum* positive (%)	*P. vivax* positive (%)	*P. malariae* positive (%)	Any species positive (%)
RDT	954	20 (2.1)	11 (1.2)	0 (0)	30 (3.1)
Microscopy	954	16 (1.7)	9 (0.9)	0 (0)	25 (2.6)
PCR	852	39 (4.6)	6 (0.7)	1 (0.1)	46 (5.4)

**Table 2:** Agreement of malaria tests among 852 OPD patients with all three test results available, Rourkela 2016–2017

RDT	Microscopy	PCR	Number (%)	Species	Report of recent malaria treatment
Positive	Negative	Negative	3 (0.4)	2 Pf & 1 Pf + Pv	2 Pf in previous month
Positive	Positive	Negative	3 (0.4)	1 Pf & 2 Pv	1 Pv 4 months before
Positive	Negative	Positive	2 (0.2)	1 Pf & 1 Pv	1 Pf in previous month
Positive	Positive	Positive	20 (2.3)	14 Pf, 5 Pv, 1 Pm*	2 Pf and 1 Pv in previous month
Negative	Positive	Negative	0		
Negative	Positive	Positive	1 (0.1)	1 Pf	None
Negative	Negative	Positive	23 (2.7)†	All Pf	None
Negative	Negative	Negative	800 (93.9)		30 (3.8%) in previous month

Abbreviations: Pf: *P. falciparum*, Pv: *P. vivax,* Pm*: P. malariae,* RDT: rapid diagnostic test, PCR: polymerase chain reaction

*P. malaria by PCR, *P. vivax* by RDT and microscopy

† Sub-patent malaria

Twenty patients were positive by all three tests (14 *P. falciparum*) with only one species discrepancy whereby RDT and microscopy detected *P. vivax* but PCR detected *P. malariae*. Mixed *Plasmodium* species (defined as more than one species detected by the same test) were identified in only one patient (*P. falciparum* and *P. vivax*). When using PCR as the gold standard, RDT had a sensitivity of 47.8% and a specificity of 99.3%; for microscopy this was 45.7 and 99.6%, respectively. By microscopy, men were more likely to be positive for malaria (3.9% vs. 1.4% among women, *p* = 0.029), whereas by PCR adults were more likely to be positive (6.5% vs. 3.0% among children, *p* = 0.036); sub-patent infections or *Plasmodium* species were not associated with age, gender, or season. Vomiting and lack of body aches were associated with malaria for all tests, e.g.*,* for RDT, vomiting was 3.80 times more likely (95% CI 1.77–8.16, *p* = 0.001) among patients with a positive RDT compared to patients without, whereas the prevalence ratio of report of body ache was 0.35 (95% CI 0.15–0.82, *p* = 0.016).

### Dengue, chikungunya, and scrub typhus

**Fig. 4:**
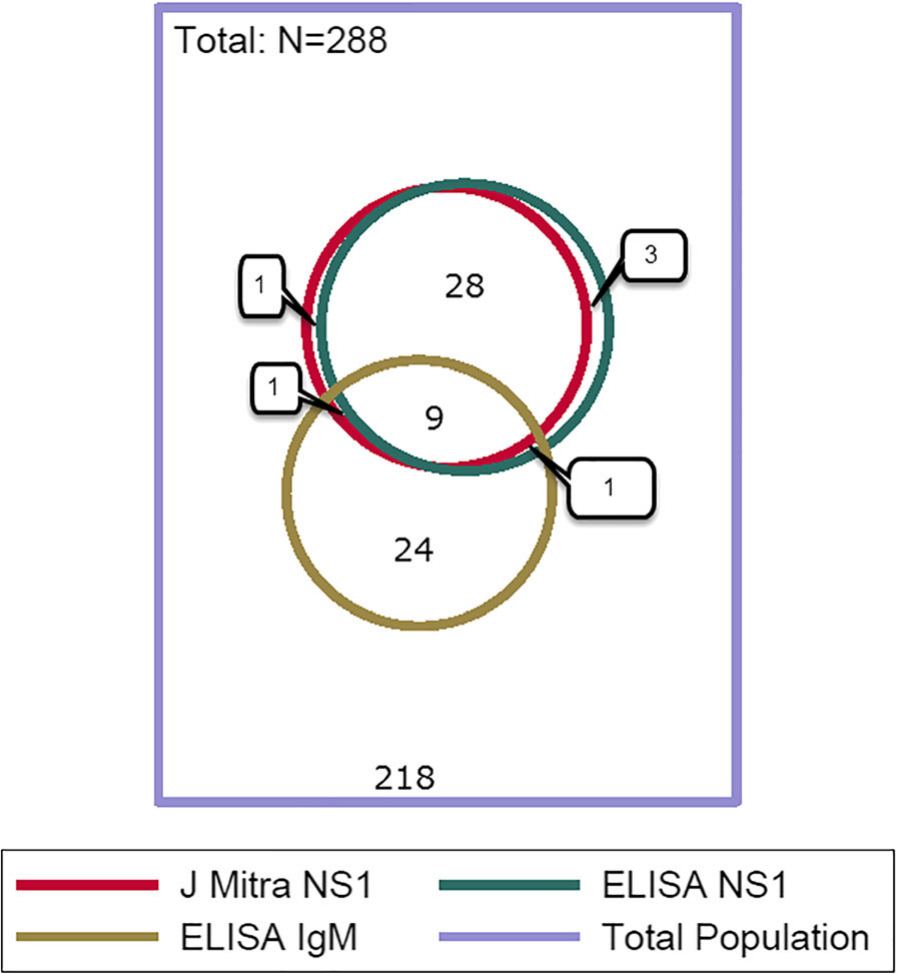
Venn diagram of select dengue test results among OPD patients, Rourkela, 2016–2017. A proportional Venn diagram representing dengue positivity among 288 study participants tested by three different assays: J. Mitra NS1 RDT, NS1 ELISA, and DENV-specific IgM ELISA. The numbers in each compartment of the Venn diagram are depicted on the graph

Seventy-four patients of 293 patients tested were positive (25.3%) for dengue by either RDT or ELISA (Table 3). Thirty-seven patients out of 285 patients tested were positive (13%) for NS1 antigen by both RDT and ELISA, and 8 patients out of 285 patients tested were positive (2.8%) for dengue virus (DENV)-specific IgM by both RDT and ELISA (Table 3). Ten patients of 288 tested were positive (3.5%) for both NS1 antigen and DENV-specific IgM by ELISA. Men were more likely to be positive than women for dengue IgM by the J. Mitra RDT (13/169 or 7.7% vs. 3/121 or 2.5%, *p* = 0.06). Rainy season was associated with a positive result for the IgM ELISA (*p* = 0.004) and NS1- specific ELISA (*p* < 0.001) tests and the J. Mitra rapid test for NS1 (*p* < 0.001). Documented fever was more common among patients who tested positive for NS1 antigen, but not for the other tests used (documented fever was 56.4% among 39 patients positive for NS1 antigen by RDT, and 24.4% among 246 negative patients, *p* < 0.001). A proportional Venn diagram (Fig. 4) shows the concordance between the dengue diagnostic test results.

**Table 3:** Detection of dengue and chikungunya by RDT and ELISA in the 293 subjects for whom sufficient plasma was available

Antigen	Type of test	Manufacturer (no. of patients tested)	Positive cases	% Positivity	Concordance between tests
Dengue NS1 (n = 293)	RDT	J. Mitra (n = 290)	39	13.4	NS1 RDT: J. Mitra vs. SD Bioline: 100% same results, *n* = 101
		SD Bioline (n = 101)	13	12.9	
	ELISA	Panbio (*n* = 288)	41	14.2	J. Mitra RDT vs. Panbio ELISA (NS1): McNemar test, *n* = 285, exact p-value 0.688
Dengue IgM (n = 293)	RDT	J. Mitra (n = 290)	16	5.5	IgM RDT: J. Mitra vs. SD Bioline: McNemar test, N = 101, exact p-value 0.625
		SD Bioline (n = 101)	1	1	
	ELISA	Panbio (n = 288)	35	12.2	J. Mitra RDT vs. Panbio ELISA (NS1): McNemar test, n = 285, exact p-value 0.688
Dengue IgG (n = 290)	RDT	J. Mitra (*n* = 290)	8	2.8	IgG RDT: J. Mitra vs. SD Bioline: 100% same results, *n* = 101
		SD Bioline (n = 101)	2	2	
Dengue NS1 or Dengue IgG or Dengue IgM	RDT or ELISA	J. Mitra or SD Bioline or Panbio (n = 293)	74	25.3	Not applicable
chikungunya IgM (*n* = 293)	RDT	J. Mitra (n = 288)	8	2.8	IgM RDT: J. Mitra vs. SD Bioline: McNemar test, *n* = 100, exact p-value 0.250
		SD Bioline (*n* = 102)	0	0	
	ELISA	Panbio (*n* = 288)	15	5.2	J. Mitra RDT vs. Panbio IgM ELISA: McNemar test, *n* = 283, exact *p*-value 0.022
	RDT or ELISA	J. Mitra or SD Bioline or Panbio (*n* = 293)	17	5.8	Not applicable

Eight patients out of 290 tested were positive (2.8%) for chikungunya virus (CHIKV)-specific IgM by either the J. Mitra or SD Bioline RDT, and 15 of 288 patients tested were positive (5.2%) by ELISA for CHIKV-specific IgM. Seventeen of 293 patients tested positive (5.8%) by either ELISA or RDT for CHIKV-specific IgM, while 6 of 285 patients tested were positive (2.1%) by both CHIKV-specific IgM RDT and ELISA (Table 3). No significant associations were detected between being positive for chikungunya and gender, age, season, or symptoms, but persons positive for chikungunya by any test were more likely to be anemic (see Additional file 1: Table S1).

Of 287 samples tested, 10 (3.5%) were positive for *Orientia tsutsugamushi*-specific IgM antibodies by ELISA. Of these, 3 samples had an O.D. value > 1 and were likely to have active or recent infection; O.D. values between 0.5 and 1, observed in 7 patients in our study, may be indicative of early or past infections (Table 4). Scrub typhus was only detected among persons > 15 years during the rainy season; patients positive for scrub typhus were more likely to be anemic (Additional file 1: Table S1).

**Table 4:** Characteristics and O.D. values of study participants positive for scrub typhus by ELISA

Subject	O.D. value	Age	Sex	Month of sample collection
IS0585	2.531	48	Male	August
IS0697	1.113	37	Female	September
IS0527	1.049	35	Female	August
IS0642	0.716	26	Female	August
IS0595	0.702	62	Male	August
IS0743	0.685	16	Male	September
IS0477	0.678	23	Male	July
IS0593	0.669	45	Female	August
IS0525	0.599	32	Male	August
IS0783	0.598	40	Male	September

*All samples tested for scrub typhus were collected between July 2016 and January 2017

### Co-infections

Of the 290 patients tested for at least three infections, 105 (36.2%) were positive for at least one pathogen, 17 (6.1%) for two, and one patient (0.4%) for three pathogens (Table 5) by any test for malaria, dengue, chikungunya, or scrub typhus. This last patient was positive for dengue, chikungunya, and *P. falciparum*. Eight out of ten patients positive for scrub typhus were also positive for dengue, chikungunya or malaria; six of these were dengue positive by any test (*p* = 0.009 for association between scrub typhus and dengue). Almost half of chikungunya positive patients (by any test) were positive for dengue, malaria or scrub typhus (8/17 or 47%); five of these were malaria positive (*p* < 0.001 for the association between malaria detected by any test and chikungunya, *N* = 280, 2 sub-patent malaria infections). No association was seen between dengue and chikungunya positives (4 co-infections with dengue positives among 17 chikungunya positive patients or 23.5%, versus 69 dengue positives among persons negative for chikungunya or 25.8%, *p* = 0.83). Patients tested for at least three infections were more likely to be adults and enrolled in the rainy season compared to patients tested for malaria only (*p* < 0.001 for both). All patients positive for multiple infections visited in the rainy season versus 93.2% of the persons with one detected infection (82/88) and 69.2% (128/185) of the patients with no infection detected (*p* < 0.001). No significant differences were detected by age, gender or symptoms compared to patients with a single or no infection; however, anemia was higher among the 17 patients positive for ≥2 infections (58.3%), compared to 26.8% in patients positive for one infection, and 22.1% in patients where none of these four infections were detected (*N* = 266, *p* = 0.018, Additional file 1: Table S1).

**Table 5:** Characteristics of 17 outpatients with multiple infections among 290 patients with at least three tests, Rourkela, 2016–2017

		Tests with positive results						
Patient	Positive for	CHIKV	DENV	Pf	Scrub typhus	Age	Gender	Month
IS0596	CHIKV, DENV, Pf	J. Mitra IgM RDT	J. Mitra IgM RDT	RDT, BS & PCR	NA	35	Male	August
IS0525	Scrub typhus, Pf	NA	NA	RDT, BS & PCR	IgM ELISA	32	Male	August
IS0642	Scrub typhus, DENV	NA	IgM ELISA & NS1 ELISA	NA	IgM ELISA	26	Female	August
IS0585	Scrub typhus, DENV	NA	IgM ELISA	NA	IgM ELISA	48	Male	August
IS0783	Scrub typhus, DENV	NA	IgM ELISA	NA	IgM ELISA	40	Male	September
IS0697	Scrub typhus, DENV	NA	NS1 ELISA	NA	IgM ELISA	37	Female	September
IS0595	Scrub typhus, DENV	NA	J. Mitra IgM RDT	NA	IgM ELISA	62	Male	August
IS0743	Scrub typhus, DENV	NA	J. Mitra IgM RDT	NA	IgM ELISA	16	Male	September
IS0477	Scrub typhus, CHIKV	IgM ELISA	NA	NA	IgM ELISA	23	Male	July
IS0742	DENV, Pf	NA	NS1 ELISA	PCR	NA	40	Female	September
IS0731	CHIKV, Pf	IgM ELISA & J. Mitra IgM RDT	NA	RDT, BS & PCR	NA	42	Male	September
IS0849	CHIKV, Pf	IgM ELISA & J. Mitra IgM RDT	NA	RDT, BS & PCR	NA	16	Male	October
IS0695	CHIKV, Pf	IgM ELISA & J. Mitra IgM RDT	NA	PCR	NA	69	Male	September
IS0674	CHIKV, Pf	IgM ELISA & J. Mitra IgM RDT	NA	RDT	NA	14	Male	September
IS0848	CHIKV, DENV	IgM ELISA	IgM ELISA	NA	NA	14	Female	October
IS0815	CHIKV, DENV	IgM ELISA	NS1 ELISA, J. Mitra IgM RDT, J. Mitra IgG RDT	NA	NA	24	Male	October
IS0586	CHIKV, DENV	J. Mitra IgM RDT	NS1 ELISA, IgM ELISA, J. Mitra NS1 RDT	NA	NA	37	Male	August

Pf: *P. falciparum*, CHIKV: chikungunya virus, DENV: Dengue virus, NA: Not applicable, RDT: rapid diagnostic test, BS: blood smear (microscopy), PCR: polymerase chain reaction

## Discussion

A summary of the few hospital-based febrile illness surveillance studies that have occurred in India is shown in Table 6. Of note is a 5-year acute febrile illness (AFI) study conducted by Manipal Centre for Virology Research (MCVR) in collaboration with the U.S. Centers for Disease Control [4]. Researchers at MCVR recruited patients at 27 hospital-based sentinel sites across India and tested them for bacterial and viral pathogens starting in June 2014. Of the 1483 in-patient and out-patient cases recruited in Odisha until July 2017, 595 (40%) were diagnosed positive for one of the tests, with malaria as the major etiology (12.4%) followed by scrub typhus (3.2%), leptospirosis (2.4%), influenza (1.4%), rotavirus (1.2%), and dengue (0.7%). The higher malaria prevalence in the MCVR study may have been observed because the RT-PCR used for parasite detection is more sensitive than regular PCR.

**Table 6:** Summary of results from six febrile illness studies from India, 2007–2017

Study	Location and year	Population and sample size	Select tests and results	Results (n, %)
Chrispal 2010	Christian medical college, Vellore; 2007	≥16 years, febrile for 5–21 days, *N* = 398 in-patients	1) Thin blood smear for malaria 2) Dengue IgM-IgG ELISA 3) Scrub typhus IgM ELISA 4) Blood culture or Typhidot for Salmonella 5) Leptospirosis IgM ELISA 6) Spotted fever IgM ELISA 7) Hantavirus IgM and IgG	Malaria (68, 17.1%) Dengue (28, 7.0%) Scrub typhus (189, 47.5%) Enteric fever (32, 8.0%) Leptospirosis (12, 3.0%) Spotted fever (7, 1.8%) Hantavirus (1, 0.3%)
Mittal 2015	Himalayan Institute of Medical Sciences, Dehradun, Dec 2012- Nov 2013	> 18 years, febrile for 5–14 days, *N* = 2547 in-patients	1) Malaria microscopy, RDT 2) Scrub typhus IgM ELISA 3) Dengue NS1/ IgM RDT 4) Leptospira IgM RDT 5) Widal Ag kit for Salmonella 6) Anti HEV IgM EIA 7) Anti HAV IgM EIA	Malaria (175, 6.8%); Scrub typhus (367, 14.4%); Dengue (956, 37.5%); Leptospirosis (0.14%); Enteric fever (424, 16.5%); Hepatitis A (1.9%); Hepatitis E (1.4%); Undetermined 11%. Mixed infections (48, 1.9%)
Abhilash 2016	Christian medical college, Vellore; Oct 2012- Sep 2013	≥16 years, febrile for 3–14 days, *N* = 1258 Both in-patients and out-patients	1) Thin blood smear for malaria 2) Dengue IgM-IgG ELISA 3) Scrub typhus IgM ELISA 4) Blood culture for Salmonella, Widal 5) Leptospirosis IgM ELISA	Malaria (131, 10.4%) Dengue (386, 30.6%) Scrub typhus (452, 35.9%) Enteric fever (47, 3.7%) Leptospirosis (8, 0.6%) Undetermined (220, 17.4%)
Morch 2017	7 hospitals in India, April 2011- Nov 2012	≥5 years, *N* = 1564, in-patients	1) Malaria PCR, RDT, microscopy 2) Dengue RDT, IgM ELISA 3) chikungunya IgM ELISA 4) Leptospirosis IgM ELISA 5) Scrub typhus IgM ELISA 6) Blood culture for bacterial infections	Malaria (268, 17%) Dengue (244, 16%) chikungunya (98, 6%) Leptospirosis (116, 7%) Scrub typhus (159, 10%) Bacteremia (124, 8%)
Robinson 2018	BJ Medical College, Pune; 2013–2015	> 6 months, fever or complaint of fever, *N* = 1725 in-patients	1) Malaria RDT, microscopy 2) Dengue NS1 ELISA, IgM 3) chikungunya IgM RDT 4) Influenza RDT 5) Leptospirosis IgM	Malaria (102, 6%) Dengue (252, 15%) chikungunya (35, 2%) Influenza (13, 0.8%) Leptospirosis (18, 1%) Mixed mosquito borne infections (23, 1%) Undetermined (965, 56%)
MCVR report 2017	27 hospital-based sentinel sites, June 2014–July 2017	*N* = 27,586 in-patients	1) PCR for Influenza 2) Dengue IgM, IgG ELISA, PCR 3) Scrub typhus IgM ELISA and PCR 4) Leptospirosis IgM ELISA, PCR, MAT 5) Malaria RDT, PCR 6) chikungunya IgM ELISA, PCR	Influenza (4118, 15%) Dengue (1898, 7%) Scrub typhus (1177, 4%) Leptospirosis (1107, 4%) Malaria (953, 3%) chikungunya (371, 1%)

In our study, we enrolled 954 patients over 12 months from January 2016 to January 2017 from an outpatient clinic in Ispat General Hospital in Rourkela, Odisha, India, who had first undergone triage for malaria by OPD staff, and we carried out diagnostic tests for febrile illnesses known to be prevalent in the area based on hospital data. We detected dengue in approximately one in eight patients by any ELISA-based diagnostic test, and positivity rates for chikungunya and scrub typhus were low (< 6%). Co-infections were commonly detected for scrub typhus and chikungunya; for example, dengue was detected in six out of the ten patients with scrub typhus and malaria detected in five of the 17 patients with chikungunya. Below we discuss each of the four diseases in depth.

### Malaria

There were 1,090,724 cases of malaria and 331 deaths reported in India in 2016, of which 449,467 cases (41.2%) and 77 deaths (23.3%) were reported from Odisha (NVBDCP). These case numbers are likely underestimates since only ~ 8% of cases may be detected by the surveillance system, as noted by the World Health Organization (W.H.O.) [24]. Even going by these low estimates, India accounts for ~ 6% of all malaria cases and 51% of *P. vivax* cases globally. Malaria transmission occurs in both urban and rural India and is especially high among tribal populations in the mountainous, inaccessible regions of eastern and northeastern India [25].

Of the three commonly used methods for malaria diagnosis (RDTs, microscopy, and PCR), microscopy remains the standard for malaria diagnosis in India. In Asian countries, males have a higher risk of malaria (as diagnosed by microscopy) compared to females (as diagnosed by microscopy) [26]; the reason is not completely clear but may relate to differences in exposure by gender. PCR-based methods are more sensitive, but require expensive equipment that is unavailable in most regions. RDTs, while less sensitive and specific than microscopy, are available and used for first-line malaria diagnosis in remote regions. Our results confirm findings of previous studies across India of higher sensitivity of PCR over microscopy when used for detection of *Plasmodium* [27, 28]. Submicroscopic malaria has been reported to be more common among older people and possibly related to the development of immunity, and also with the recent use/suboptimal use of antimalarial drugs [29]. Three patients were positive by RDT and negative by microscopy and PCR: the antigens produced by recently-cleared malaria parasites can persist in the blood after treatment for a period of time, explaining the positive RDT in the presence of a negative PCR test [30]. In our previous treatment studies, we have noted that for *P. falciparum* (although not for *P. vivax*), some RDTs can be positive up to 28 days post treatment, whereas the PCR test result for the same patient was negative in 1 week. No patient identified as *Plasmodium* positive by microscopy was negative by both RDT and PCR. However, there were 23 sub-patent infections among the 46 patients positive for *Plasmodium* by PCR. Sub-patent infections are of concern, because malaria infections not detected by microscopy or RDT may not be treated appropriately; patients may continue to carry the parasite and act as a pool for further transmission. Overall, our finding that 54 out of 954 patients (5.7%) were positive for *P. falciparum*, *P. vivax,* or *P. malariae* by at least one diagnostic method despite all 954 patients having undergone malaria triage in the OPD leads us to recommend the use of malaria diagnostic test(s) rather than relying upon patient symptoms.

### Dengue

The incidence of dengue has grown dramatically around the world in recent decades. The WHO predicts an incidence of 50–100 dengue million cases and ~ 20,000 dengue-related deaths per year, and nearly 40% of the world’s population lives in areas with a risk of DENV transmission [31]. India is estimated to contribute 34% of the total global burden of dengue [32], and suffered several outbreaks of severe dengue over the last few years [33–36]. There were 129,166 reported cases with 245 deaths in 2016 (NVBDCP). Dengue is endemic in Odisha since 2010, and there has been a four-fold increase in the number of reported cases, from 2450 in 2015 to 8380 in 2016, accounting for 6.5% of the total cases in India [35].

Dengue diagnosis in our study was carried out by detection of either the NS1 antigen or antibodies specific to DENV envelope proteins. We used two techniques for detection of DENV: RDTs to detect NS1, DENV-specific IgG and IgM, and ELISA targeting either NS1 or DENV-specific IgM. NS1 antigen is present in the bloodstream immediately after onset of fever for up to 18 days, and detection of NS1 can thus be used as a proxy for early infection. DENV-specific IgM antibodies may be present in the serum as early as 3–5 days following onset of fever, and persist for several months, in the case of a primary infection, but appear earlier in a secondary infection. IgG antibodies appear 14 days after a primary dengue infection, and can persist for life, but appear within the first couple of days following onset of symptoms in a secondary infection.

We observed a 100% concordance of Dengue NS1 positivity between 101 samples tested by RDTs from two different manufacturers (J. Mitra and SD Bioline) in India, with a positivity rate of ~ 13%. A slightly larger but comparable number of samples (~ 14.2%) were positive when tested by ELISA for NS1 antigen. Four samples were NS1 antigen positive by ELISA but not RDT, and two samples were positive by RDT but not ELISA. There was a significant difference in the number of samples positive by ELISA for Dengue NS1 versus DENV-specific IgM, as expected since these methods test chronologically different stages of the infection, with NS1 antigen presence in the serum preceding DENV-specific IgM by 3–5 days. However, there were significantly fewer samples identified as positive for DENV-specific IgM by RDT than ELISA (20%), which may be due to the increased sensitivity of the capture ELISA IgM over the RDT. Only 2 samples were positive for DENV-specific IgG by the SD Bioline RDT but they were also positive by the J. Mitra RDT. There was also a large variation in positivity in our dengue test results, ranging from 1 to 14.2% by any single test, and 25.3% positive across all dengue tests combined, so it is likely that some positives will be missed if just one diagnostic test is used. Dengue prevalence rates varied from 7 to 38% across other hospital-based febrile illness studies in India and depending upon the assay used; the highest prevalence was observed in a study that used RDTs for detection of DENV **(**Table 6**).**

### Chikungunya

Chikungunya transmission is concentrated in Southeast Asia, the Americas, Pacific Islands, and Africa. The virus has a high infection rate, with up to 75% of a population infected in areas of virus circulation [37]. Early documented outbreaks of chikungunya in India in the 1960s and 1970s were followed by a major outbreak in Kenya in 2004, which spread to the Indian subcontinent and southeast Asia by 2010 [8, 38]. Since then, there has been continuous transmission of chikungunya in India [8, 39], with an increase in number of cases in India from 27,553 in 2015 to 64,057 in 2016, although in Odisha the number of cases decreased from 81 to 51 during the same period (NVBDCP).

A definitive diagnosis can be accomplished using three laboratory tests: virus isolation, serology, and PCR. We used both an immunochromatography-based RDT as well as an ELISA assay to measure chikungunya-specific IgM levels in patient plasma. It is important to note that chikungunya-specific IgM is usually detected in the plasma from day 4–7 following the onset of symptoms, so we may have missed early infections. In our study, 2.8% patients tested were positive for chikungunya-specific IgM by the RDT (J. Mitra and Co.), but twice as many (5.2%) were found to be positive by the more sensitive ELISA based assay. We did not have the resources to confirm the test results with virus culture and RT-PCR, so it is unclear which test result is more reliable, and thus, further examination of these results is warranted. Similar proportions of chikungunya IgM-positive individuals (2–6%) were observed in other hospital-based studies from India (Table 6) [4, 5, 11].

### Scrub typhus

Scrub typhus is a re-emerging infection in India in that its prevalence has been increasing in recent years across various parts of the country [11]. It is underreported [11, 40], and prevalence data are not easily available as routine field and hospital-based surveillance has only recently begun [41]. It rose to prominence as a dominant cause of fever among soldiers along the Indo-Burmese border during World War II, and the Indo-Pakistan border in the 1990s [42]. India belongs to the ‘tsutsugamushi triangle’, the postulated zone of highest prevalence of scrub typhus cases [42], and the illness has been found to be prevalent ubiquitously across the country [40, 43–47].

Scrub typhus diagnosis may be carried out by one or more of the following methods: IgM and IgG ELISA to test for the presence of anti-*O. tsutsugamushi* antibodies in patient serum, and PCR targeting *O. tsutsugamushi* antigenic genes. We used the Scrub Typhus Detect ELISA kit from InBios international to identify IgM antibodies targeting the *O. tsutsugamushi*-derived recombinant 56-kDa antigen, as an indicator of exposure to the bacterium. We observed a positive result in 10/287 (3.5%) patient sera tested. Other hospital-based surveillance studies in India have identified scrub typhus as a frequent etiology of acute febrile illness, with frequencies varying from 9 to 48% [4, 11, 44, 48, 49] (Table 6). We were unable to test for the presence of *Orientia* bacteria by PCR and did not collect convalescent sera from patients to test an increase in antibody titers by IFA. We may also have missed some scrub typhus cases if the plasma sample was collected too early in the infection. Eschar was not observed in any patients.

#### Co-infections

DENV and CHIKV are both transmitted by the *Aedes* mosquito, leading to significant overlap in areas of circulation of these viruses. Several studies report co-infections of dengue and chikungunya in India [15, 50–52]. In our study, we found a statistically significant association between chikungunya and malaria, and between scrub typhus and dengue, but not between chikungunya and dengue. The number of co-infections with malaria as one of the infections is likely an underestimate due to our patient pool having undergone triage for malaria on the basis of presumptive malaria symptoms of high fever, chills and rigor.

#### Anemia

Anemia was associated with chikungunya, scrub typhus, and multiple infections (Additional file 1: Table S1). Although anemia is a frequent reported effect of malaria, only a borderline association with malaria as diagnosed by RDT was detected. It is possible that anemia was not associated with malaria because our study sample set was derived from an outpatient population with a high prevalence of anemia (39%), and the etiology of anemia is diverse.

#### Future studies

Our pilot study testing for four common infectious diseases could be expanded to include additional components. For example, conducting viral culture or RT-PCR-based assays would increase the robustness of our studies. Apart from malaria, dengue, chikungunya, and scrub typhus, there are other etiologies for febrile illness prevalent in Odisha that we could test for in the future, including the bacterial illnesses leptospirosis, melioidosis, and typhoid fever, and the viral illnesses influenza and Japanese encephalitis.

## Conclusions

Of the three infectious diseases (dengue, chikungunya, and scrub typhus) we tested patients for after initial triage for malaria cases by the OPD, dengue was the most common infection detected (25.3% of all patients tested by either RDT or ELISA). Our results suggest that using more than one dengue diagnostic test is important since available diagnostic tests have different sensitivities and capture different time points in an infection. Our results also recommend using diagnostic tests to identify the malaria parasite rather than relying upon patient symptoms. Although other infections were less common, physicians should keep the possibility of co-morbidity in mind when treating patients. Routine febrile illness surveillance is required to accurately establish the prevalence of these infections in this region, to offer timely treatment, implement appropriate methods of control, and ultimately inform policy.

## Additional file

Additional file 1: **Table S1.** A. Anemia of participants for each diagnostic test. B. Anemia of participants according to number of infections*. The association of anemia with patients tested for malaria, dengue, chikungunya and scrub typhus by every diagnostic test used is presented in supplemental Table 1, and the association between anemia and number of infections detected in a patient is presented in supplemental Table 2. (DOCX 29 kb)

## Data Availability

Data generated and analyzed during this study are available through this published article and its supplementary information files. In addition, data have been submitted to the EuPathDB clinical database ClinEpiDB (https://clinepidb.org/), part of the EuPathDB project [53].
