# Supplementary material for: Dengue, chikungunya, and scrub typhus are important etiologies of non-malarial febrile illness in Rourkela, Odisha, India
Source: BMC Infect Dis. 2019 Jul 3;19:572. doi: 10.1186/s12879-019-4161-6 (PMC6607595; doi:10.1186/s12879-019-4161-6)
Supplement: Supplementary file 1 — Table S1. A. Anemia of participants for each diagnostic test. B. Anemia of participants according to number of infections*. The association of anemia with patients tested for malaria, dengue, chikungunya and scrub typhus by every diagnostic test used is presented in supplemental Table 1, and the association between anemia and number of infections detected in a patient is presented in supplemental Table 2. (DOCX 29 kb) [file 12879_2019_4161_MOESM1_ESM.docx]

**Supplemental Table 1. A. Anemia of participants for each diagnostic test. B. Anemia of participants according to number of infections***

| **A. Anemia by diagnostic test** | | | | |
| --- | --- | --- | --- | --- |
| **Infection** | **Diagnostic test** | **No. with anemia/total no. positive for the test (%)** | **No. with anemia/total no. negative for test (%)** | **P-value** |
| Malaria | RDT | 12/21 (57.1) | 329/857 (38.4) | 0.08 |
|  | Microscopy | 7/16 (43.8) | 334/862 (38.8) | 0.68 |
|  | PCR | 12/36 (33.3) | 281/759 (37.0) | 0.65 |
|  | Any | 15/41 (36.6) | 278/754 (36.9) | 0.97 |
| Dengue | RDT NS1 | 12/38 (31.6) | 58/228 (25.4) | 0.43 |
|  | SD Bioline NS1 | 6/12 (50.0) | 24/82 (29.3) | 0.15 |
|  | Elisa NS1 | 12/40 (30.0) | 54/224 (24.1) | 0.43 |
|  | J. Mitra † | 3/16 (18.8) | 67/250 (26.8) | 0.15 |
|  | SD Bioline † | 0/3 | 30/91 (33.0) | 0.48 |
|  | Any | 21/69 (30.4) | 46/194 (23.7) | 0.27 |
| Chikungunya | J. Mitra IgM | 3/6 (50.0) | 67/258 (26.0) | 0.19 |
|  | SD Bioline | 0 | 30/95 (31.6) |  |
|  | IgM | 5/12 (41.7) | 61/252 (24.2) | 0.17 |
|  | Any | 7/14 (50.0) | 60/246 (24.4) | 0.03 |
| Scrub typhus | IgM | 4/7 (57.1) | 62/256 (24.2) | 0.047 |

| **B. Anemia by number of infections** | | |
| --- | --- | --- |
| **No. of infections** | **No. with anemia/number of people in infection group** | **P-value** |
| 0 infection | 38/172 (22.1) |  |
| 1 infection | 22/82 (26.8) |  |
| >1 infection | 7/12 (58.3) | 0.018 |

*Anemia available for 878 participants. Age and gender appropriate definition: Hemoglobin <11 g/dl if age <5 years, <11.5 g/dl if age ≥5 and <12 years, <12 g/dl if age >12 and age<15, <12 g/dl if age ≥15 and female, and <13 g/dl if age ≥15 years and male

† IgM or IgG or both
